# Genomic DNA Copy Number Aberrations, Histological Diagnosis, Oral Subsite and Aneuploidy in OPMDs/OSCCs

**DOI:** 10.1371/journal.pone.0142294

**Published:** 2015-11-05

**Authors:** Patrizio Castagnola, Gabriele Zoppoli, Sergio Gandolfo, Massimiliano Monticone, Davide Malacarne, Gabriella Cirmena, David Brown, Cinzia Aiello, Massimo Maffei, Roberto Marino, Walter Giaretti, Monica Pentenero

**Affiliations:** 1 IRCCS AOU - San Martino -IST, Genoa, Italy; 2 Department of Internal Medicine, University of Genoa, Genoa, Italy; 3 Department of Oncology, Oral Medicine and Oral Oncology Unit, University of Turin, Turin, Italy; 4 J.-C. Heuson Breast Cancer Translational Research Laboratory, Institut Jules Bordet, Université Libre de Bruxelles, Brussels, Belgium; University Medicine Greifswald, GERMANY

## Abstract

Oral potentially malignant disorders (OPMDs) characterized by the presence of dysplasia and DNA copy number aberrations (CNAs), may reflect chromosomal instability (CIN) and predispose to oral squamous cell carcinoma (OSCC). Early detection of OPMDs with such characteristics may play a crucial role in OSCC prevention. The aim of this study was to explore the relationship between CNAs, histological diagnosis, oral subsite and aneuploidy in OPMDs/OSCCs. Samples from OPMDs and OSCCs were processed by high-resolution DNA flow cytometry (hr DNA-FCM) to determine the relative nuclear DNA content. Additionally, CNAs were obtained for a subset of these samples by genome-wide array comparative genomic hybridization (aCGH) using DNA extracted from either diploid or aneuploid nuclei suspension sorted by FCM. Our study shows that: i) aneuploidy, global genomic imbalance (measured as the total number of CNAs) and specific focal CNAs occur early in the development of oral cancer and become more frequent at later stages; ii) OPMDs limited to tongue (TNG) mucosa display a higher frequency of aneuploidy compared to OPMDs confined to buccal mucosa (BM) as measured by DNA-FCM; iii) TNG OPMDs/OSCCs show peculiar features of CIN compared to BM OPMDs/OSCCs given the preferential association with total broad and specific focal CNA gains. Follow-up studies are warranted to establish whether the presence of DNA aneuploidy and specific focal or broad CNAs may predict cancer development in non-dysplastic OPMDs.

## Introduction

Oral cancer is often diagnosed at a late stage and therefore as a result is characterized by poor prognosis. The five-year survival rate of this disease is below 50% [1, 2] and in this context, early detection and therapeutic intervention are crucial [3]. Oral cancer may develop from oral potentially malignant disorders (OPMDs), and the presence of dysplasia in OPMDs has been reported as a risk factor for malignant transformation [4]. However, assessment of dysplasia based on the WHO classification is subjective and recently led to the proposal of an improved method [5]. Furthermore, oral cancer may also develop from non-dysplastic OPMDs (ND-OPMDs) or even in normal looking oral mucosa fields as well as in the oral mucosa of patients with no history of a previous OPMD [6, 7]. To date, there have been no reports of molecular markers that are able to predict the progression of either normal appearing mucosa or of these disorders to invasive cancer [8–12]. However, several studies have highlighted biomarkers associated with DNA ploidy [13–18], loss of heterozygosity (LOH) [19], expression of specific genes involved in cell cycle, growth factor signaling and tumor suppressor genes [20]. Genomic alterations such as aneuploidy, DNA copy number aberrations (CNAs) and point mutations are all markers of genotoxic exposure and DNA damage. Several genetic mechanisms, including the presence of an aberrant number of centrosomes and the missegregation of single chromosomes may promote chromosomal instability (CIN) [21–23], which is considered a driver of aneuploidy [24] and fosters tumor progression [25]. However, several studies have shown that aneuploidy itself may promote CIN [23, 26]. The association between CNAs, DNA ploidy, site of origin of the OPMD/oral cancer within the oral mucosa, and the histology of the OPMD/cancer are not yet fully understood. Thus, it is still a challenge to predict the risk of progression from OPMD to neoplasia [27].

In the present prospective study, which includes patients with OPMDs and oral squamous cell carcinomas (OSCCs), we have addressed the analysis of these correlations through the use of DNA ploidy and CNAs as obtained by high-resolution DNA flow cytometry (hr DNA-FCM) and array comparative genomic hybridization (aCGH).

## Materials and Methods

### Patients and tissue specimens

Patients with OPMDs or OSCCs were enrolled in the study by the Oral Medicine and Oral Oncology Unit of the University of Turin at the A.O.U. S. Luigi Gonzaga (Orbassano-Turin) and in the Department of Otolaryngology, "IRCCS A.O.U. San Martino—IST" in Genoa. Written informed consent was obtained from all the enrolled patients as requested by the Institutional Ethics Committees (A.O.U. S. Luigi Gonzaga Prot. N. 11780 and San Martino Hospital Prot. N. 1084), which specifically approved this study. Declaration of Helsinki protocols were followed in designing the study.

Histological evidence of one or more OPMDs (homogeneous and non-homogeneous leukoplakias, erythroplakias and erythroleukoplakias) or of OSCC was considered inclusion criteria, while patients with history of previous oropharyngeal neoplasia were excluded from the study.

Incisional biopsies and micro-biopsies (carried out by means of a curette) of each OPMD/OSCC were obtained and performed as previously reported [28]. In some cases, multiple biopsies were performed on a single OPMD or OSCC.

Histological diagnosis was performed according to WHO guidelines by a specially trained pathologist [28, 29]. The dysplastic OPMD (D-OPMD) cases included all degrees of dysplasia, which were recorded in the diagnosis.

Bioptic samples for both FCM and aCGH analyses were either immediately processed or stored at -20°C and processed at a later time.

Table 1 reports the anatomical subsite distribution of OPMDs/OSCCs in our cohort of 292 patients.

**Table 1:** Number of bioptic samples used to isolate the nuclei suspension and perform hr DNA-FCM analysis subdivided by oral mucosa subsite and histology.

Oral mucosa subsite	Histology		
	ND-OPMD^1^	D-OPMD^2^	OSCC^3^
Buccal mucosa	183	19	16
Tongue	67	22	54
Floor of the mouth	21	7	7
Gum	49	5	15
Soft palate	10	4	6
Lip	2	0	0
Hard palate	23	2	10

^1^ Non-dysplastic oral potentially malignant disorder.

^2^ Dysplastic oral potentially malignant disorder.

^3^ Oral squamous cell carcinoma, OSCC.

Table 2 reports the number of patients enrolled in the study as well as the histology and number of oral mucosa subsites **analyzed by** FCM and aCGH. Some patients showed multiple OPMDs/OSCCs at presentation which were located on single or multiple oral subsites.

**Table 2:** Patients enrolled in the study, type and number of oral mucosa subsites that underwent biopsy and were analyzed by hr DNA-FCM and aCGH.

	hr DNA-FCM analysis	
Histology	Oral mucosa subsites	Patients
ND-OPMD^1^	224	
D-OPMD^2^	34	292^a^
OSCC^3^	73	
	aCGH analysis	
Histology	Oral mucosa subsites	Patients
ND-OPMD	46	
D-OPMD	15	94^b^ (99^c^)
OSCC	33	

^1^ Non-dysplastic oral potentially malignant disorder.

^2^ Dysplastic oral potentially malignant disorder

^3^ Oral squamous cell carcinoma, OSCC.

^a^ 155 females and 137 males; median age 66.9 (range 19.8–93.8) and 60.3 (range 18.5–86.3) years, respectively. Three hundred thirty one OPMDs/OSCCs oral subsites of 292 patients were sampled by single or multiple biopsies yielding 522 bioptic samples (see also Table 1). The nuclei suspensions of a subset of 145 bioptic samples obtained from 99 oral mucosa subsites of 99 of these 292 patients were also processed for DNA extraction and subsequent aCGH analysis. Therefore, the DNA index (DI) was also measured for each of the 145 DNA samples processed for aCGH. A single OPMD or OSCC for each patient was examined by aCGH.

^b^ 52 females and 42 males; median age 70.5 (range 19.8–93.8) and 60.3 (range 18.5–86.3) years, respectively.

^C^ The bioptic samples of 5 patients were removed from the statistical analysis because they did not comply with the aCGH quality control criteria.

### Processing of bioptic samples for hr DNA-FCM analysis and sorting

DAPI stained nuclei suspensions from bioptic samples were obtained as described by Otto et al. [30] as per previously reported modifications [31]. DNA content histograms to evaluate the DNA Index (DI) were obtained from these DAPI stained nuclei suspensions by hr DNA-FCM performed as previously reported [31]. When DNA aneuploid sublines (DI ≠ 1) were detected, these were sorted using a Cyflow Space FCM equipped with a PPCS unit (Partec GmbH, Muenster, Germany) at a purity of about 99% [31].

### DNA extraction and amplification

The Archive Pure DNA kit (5 Prime, Hamburg, Germany) was used to perform DNA extraction. A whole genomic DNA amplification was performed using the Enzo BioScore^™^ Screening and Amplification Kit (Enzo life sciences, Farmingdale, NY, USA) or the GenomePlex Whole Genome Amplification Kit WGA2 (Sigma-Aldrich, St. Louis, MO), according to the manufacturers' instructions. This amplification step was necessary in order to obtain enough DNA to perform the aCGH analysis [32].

### DNA copy number analysis by aCGH

DNA copy number aberrations were determined using high-density aCGH (2x105K, n = 76 DNA samples or 4x180K, n = 69 DNA samples) (Agilent Technologies, Palo Alto, CA, USA). These 145 DNA samples were obtained from 99 patients that in some cases underwent multiple biopsies (see also the paragraph regarding patients and tissue specimens and Table 2). For DNA labeling and assessment of DNA labeling efficiency, 0.8 μg of amplified test and reference (female or male normal genomic DNA: Promega, Madison, WI) were labeled using the Sure Tag DNA labeling kit (Agilent Technologies, Palo Alto, CA, USA) with Cy5-dUTP and Cy3-dUTP, respectively, according to the CGH Enzymatic Labeling Kit Protocol v.7.2 (Agilent Technologies, Palo Alto, CA, USA). Unincorporated nucleotides were then removed using centrifugal filters (Amicon Ultra 0.5ml, Merck Millipore, Merck KGaA, Darmstadt, Germany) according to the manufacturer’s instructions. Quality analysis and quantification of labeled DNA were performed by NanoDrop^®^ ND-1000 (Thermo Scientific, Inc.) spectrophotometry measuring A260 (for DNA), A550 (for Cy5) and A649 (for Cy3) to evaluate yield, degree of labeling and specific activity. To perform array hybridization and scanning, Cy5-labeled tumor DNA was mixed with an equivalent amount of Cy3-labeled reference DNA. Repetitive sequences were blocked with human Cot-1 DNA (Invitrogen^™^, Thermo Scientific, Inc.) and samples were hybridized with Oligo aCGH/ChIP-on-chip Hybridization Kit onto the SurePrint G3 Human CGH 4x180K Microarrays, or Human Genome CGH 105A 2x105k Microarrays (Agilent Technologies, Palo Alto, CA, USA) according to the manufacturer’s instructions followed by hybridization at 65°C for 24 hours in a rotating hybridization oven (Agilent Technologies, Palo Alto, CA, USA) at 20rpm. Microarray slides were washed according to the manufacturer’s instructions and scanned using an Agilent Microarray Scanner (G2505C). The resulting images were then processed using the Feature Extraction software v11.01.1 (Agilent Technologies, Palo Alto, CA, USA). *Log10* ratios extracted by the Agilent feature extraction software were imported in R, averaged over probe replicates using the *R/* Bioconductor package *limma*, and back-transformed into log2 scale. Shared probes between the two microarray designs were retained for downstream analysis. Arrays were discarded when the derivative Log Ratio spread (dLRs) was > 0.35, although individual files with borderline higher values were still included upon visual inspection of the raw *log2* ratio genome plots. After mapping the probe location to the NCBI37/hg19 build of the human reference genome, the *log2* ratios were smoothed by outlier winsorization using the median absolute deviation (MAD) and segmented by penalized least square regression [33] using a heuristically chosen value of γx = 40 which optimized the number of segments per sample though without leading to excessive information loss. These analysis steps were carried out using the R/Bioconductor package *copynumber* [33]. The segmented data were visually inspected for over/under segmentation. The processed data were then used to identify broad and focal CNAs using the GISTIC2.0 [34] tool on the publicly available GenePattern server (http://genepattern.broadinstitute.org/gp/pages/login.jsf) with default parameters. Thresholds for calling gain and loss were set to ± 0.3 considering that a fully clonal hemizygous deletion in a diploid genome background with a cancer cell fraction of ~ 50% would result in a *log2* ratio ≈ -0.4, whereas a single copy gain in a similar context would result in a *log2* ratio ≈ 0.3.

$$Log_{2}\;ratio=log_{2}\left(\frac{\alpha q_{t}+2\;\left(1-\alpha \right)}{\alpha D+2\;\left(1-\alpha \right)}\right)$$

$$Log_{2}\;ratio\;\left\{hemizygous\;deletion\right\}=\;log_{2}\left(\frac{.5\;\times \;1+2\;\left(1-\;.5\right)}{.5\;\times \;2+2\;\left(1-\;.5\right)}\right)=\;-0.41$$

$$Log_{2}\;ratio\;\left\{single\;copy\;gain\right\}=\;log_{2}\left(\frac{.5\;\times \;3+2\;\left(1-\;.5\right)}{.5\;\times \;2+2\;\left(1-\;.5\right)}\right)=\;0.32$$

where α is the cancer cell fraction, *q*_*t*_ is the total copy number and *D* is the ploidy.

Only focal CNAs with a q-value < 0.25 and broad CNAs with a q-value < 0.3 were considered relevant for further analysis [33, 35]. Raw and processed data are available in GEO (http://www.ncbi.nlm.nih.gov/geo/) under the accession number GSE66136.

### Statistical analysis

All sets of data from a specific oral mucosa subsite in a given patient with at least one valid OPMD/OSCC sample were processed according to the criteria described below and then used as a statistical unit in all analyses.

With reference to the histological status, in the presence of multiple tissue samples from the same statistical unit, the most severe diagnosis was taken as the reference standard for further evaluation. Therefore, we referred to each statistical unit as either OPMD (non dysplastic or dysplastic) or OSCC.

A similar criterion was used to assign the ploidy to any given OPMD/OSCC in our cohort. Therefore, the presence of a single DNA aneuploid sample among multiple samples from the same patient subsite sufficed to assign a DNA aneuploid status to the respective OPMD/OSCC.

Structural aberrations of the genomic DNA include copy number gains or losses. It has not yet been fully established whether focal or broad (more than a half a chromosome arm) CNAs differ in their ability to cause genetic instability and to increase the risk of developing cancer in the oral mucosa. Therefore, we used both focal and broad CNAs detected by the GISTIC2.0 analysis as an index of the extent of genomic DNA damage. In particular, we calculated the total number of focal CNA gains (TFG) and losses (TFL) which occurred within each OPMD/OSCC by calculating the sum of the chromosomal cytobands affected by one or more focal CNA gain or loss events. Likewise, each chromosomal region corresponding to more than half a chromosomal arm, showing a copy number gain or loss in at least one sample from a given OPMD/OSCC contributed once to the total number of broad gains (TBG) and total broad losses (TBL), respectively. By using this approach a single value of focal gain, focal loss, broad gain and broad loss aberration was obtained for each OPMD/OSCC.

In order to investigate the relationships among CNAs, DNA ploidy and histology, we performed the analysis of single or stratified 2 by 2 contingency tables using Fisher's exact test.

When indicated, the Breslow-Day (BD) test was applied to assess the homogeneity of the odds ratios for stratified 2 by 2 tables and a two-tailed Mann—Whitney (MW) U test was applied to compare count data from two groups.

Cohen’s Kappa coefficient was calculated to evaluate the co-occurrence of CNAs in OPMD/OSCC samples.

To address the problem of multiple testing, we calculated false discovery rates (FDR) q-values downstream from the selection of CNAs made by GISTIC2.0, as per Carlson et al. [36] http://research.microsoft.com/en-us/um/redmond/projects/MSCompBio/FalseDiscoveryRate/default.aspx.

This method was specifically designed for 2 by 2 contingency tables [36]. In the present analysis we chose to filter out the tests that can be proven to be irrelevant [36]. An FDR q-value method [37–39] was also applied for multiple testing corrections in the evaluation of the relationships among DNA aneuploidy, histology, total CNAs and oral subsite.

We adopted an arbitrary q-value threshold of 0.1 (or 10%) based on previous reports [40, 41] so as to keep the rate of false-positive findings as low as possible and hence to increase our ability to identify biologically relevant associations.

## Results

### Relationship between DNA ploidy, histology and oral mucosa subsite in OPMDs and OSCCs

To assess the relationship between the DNA ploidy status (DNA diploid, DI = 1 or DNA aneuploid, DI ≠ 1 and histology in OPMDs and OSCCs, all bioptic samples obtained from the enrolled patients were processed to obtain nuclei suspensions and analyzed by hr DNA-FCM to measure the DI values.

**Fig 1:**
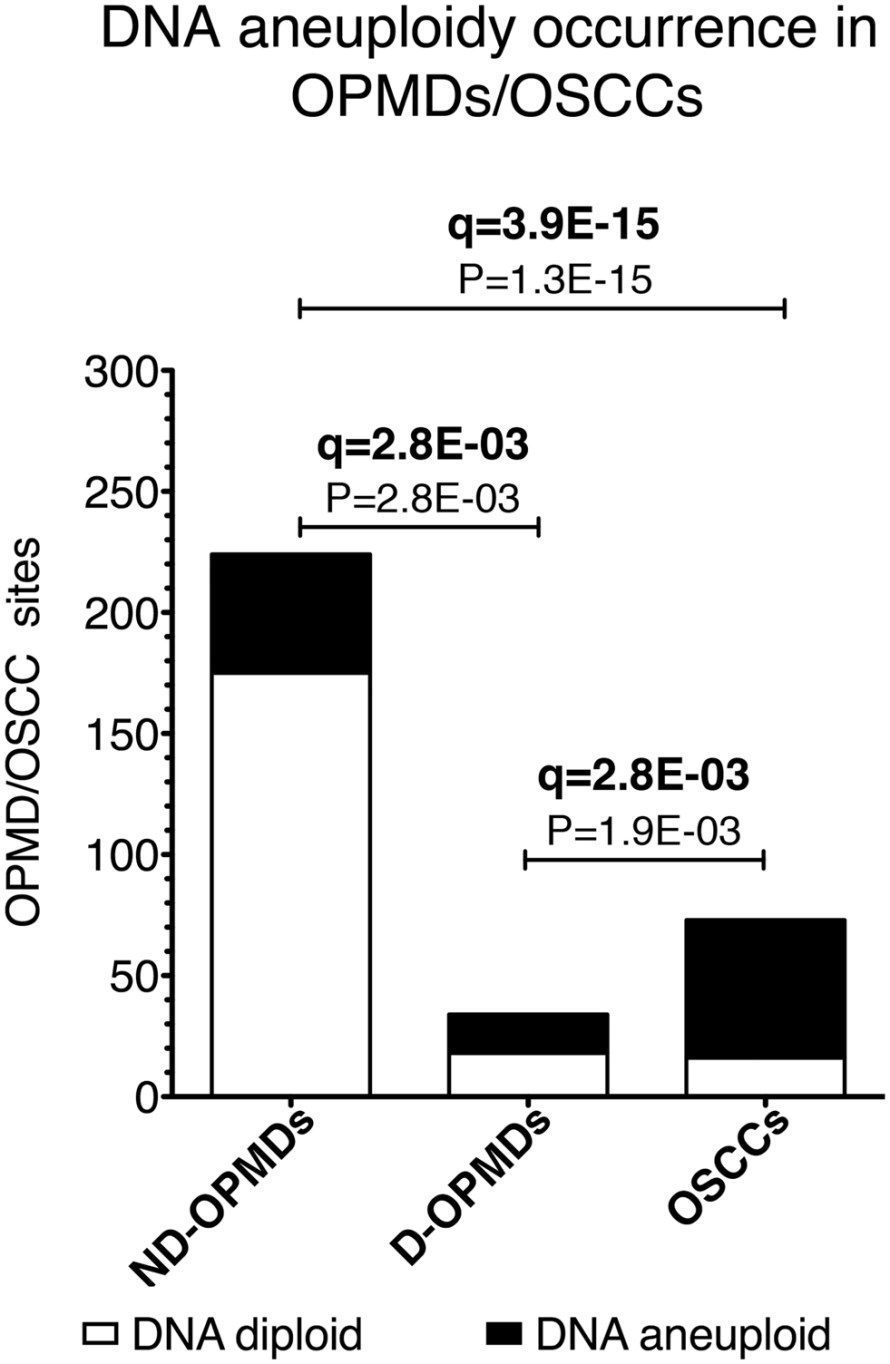
Relationship between DNA aneuploidy and histological diagnosis in OPMDs/OSCCs. DNA diploid oral potentially malignant disorders (OPMDs) and oral squamous cell carcinomas (OSCCs) are shown in white stacked bars, while DNA aneuploid OPMDs/OSCCs are shown in black stacked bars. Non-dysplastic oral potentially malignant disorder (ND-OPMD); dysplastic oral potentially malignant disorder (D-OPMD). Significant P-values (P < 0.05) are shown. The FDR q-value method was applied for multiple testing (n = 4) correction; q-values < 0.1 are indicated in bold. N = 331 OPMDs/OSCCs; N = 224 ND-OPMDs; N = 34 D-OPMDs; N = 73 OSCCs.

Fig 1 shows that the DNA aneuploidy/DNA diploid ratio was lowest in ND-OPMDs, intermediate in D-OPMDs and highest in OSCCs (ND-OPMD vs. D-OPMD, P = 2.8E-03, q = 2.8E-03; ND-OPMD vs. OSCC, P = 1.3E-15, q = 3.9E-15; D-OPMD vs. OSCC, P = 1.9E-03, q = 2.8E-03).

**Fig 2:**
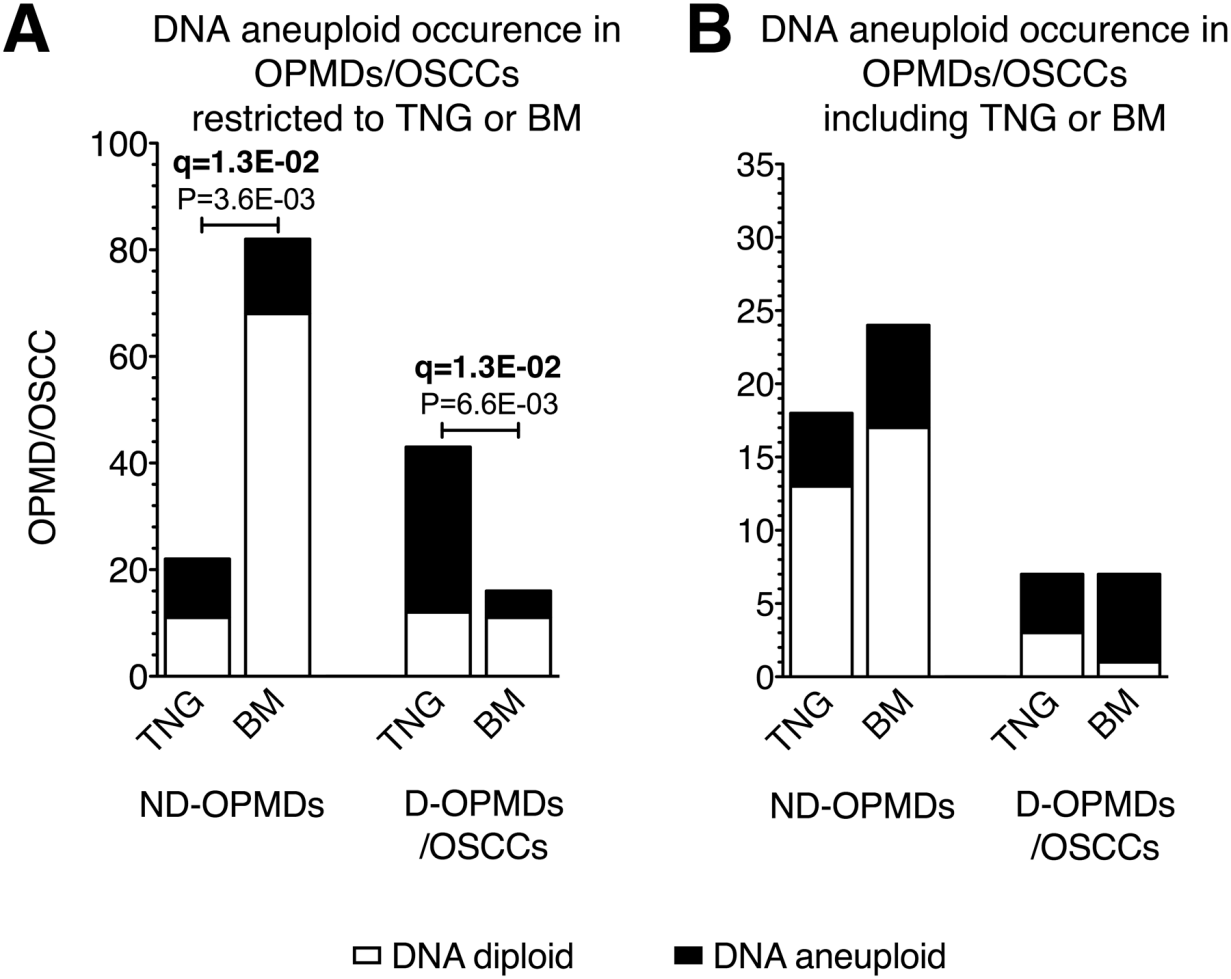
Relationship between DNA aneuploidy, histological diagnosis in OPMDs/OSCCs and oral subsite TNG or BM. (A) shows the results of the analysis of oral potentially malignant disorders (OPMDs) and oral squamous cell carcinomas (OSCCs) limited to the tongue (TNG) or buccal mucosa (BM) mucosa; (B) shows the results of the analysis of TNG or BM from patients with OPMDs/OSCCs in multiple oral subsites. Non-dysplastic OPMDs (ND-OPMDs); dysplastic OPMDs (D-OPMDs). DNA diploid oral mucosa sites are shown in white stacked bars, while DNA aneuploid oral mucosa sites are shown in black stacked bars. Significant P-values (P < 0.05) are indicated. The FDR q-value method was applied for multiple testing (n = 4) correction; q-values < 0.1 are indicated in bold. (A) N = 163; N = 22 TNG and N = 82 BM ND-OPMDs; N = 43 TNG and N = 16 BM D-OPMDs/OSCCs. (B) N = 56; N = 18 including TNG and N = 24 including BM ND-OPMDs; N = 7 including TNG and N = 7 including BM D-OPMDs/OSCCs.

When we tested the hypothesis that the occurrence of DNA aneuploidy differed between tongue (TNG) and buccal mucosa (BM) OPMDs/OSCCs, we found that the proportion of DNA aneuploidy was higher in ND-OPMDs limited to TNG mucosa (i.e., from patients with OPMDs/ OSCCs only in the TNG mucosa) compared to ND-OPMDs limited to the BM (P = 3.6 E-03, OR 4.9, CI 1.6–15.2, q = 1.3E-02) and in D-OPMD/OSCC limited to the TNG mucosa compared to D-OPMD/OSCC limited to the BM (P = 6.6E-03, OR 5.7, CI 1.4–24, q = 1.3E-02) (Fig 2A). A comparison of samples from patients who had OPMDs/OSCCs in multiple oral subsites showed that the occurrence of DNA aneuploidy was similar between TNG and BM (Fig 2B).

### Relationship between CNAs, DNA ploidy and histological diagnosis in OPMDs and OSCCs

To explore the association between CNAs and histology in OPMDs and OSCCs and to verify the expected association between CNAs and DNA aneuploidy, the genomic DNA obtained from a subset of mucosa samples of the enrolled patients underwent whole genome amplification, and was analyzed using aCGH (Table 2).

Eighty significant CNAs in our sample set were identified by GISTIC2.0 (see S1 and S2 Tables). The absence or presence of these CNAs versus ploidy status (DI = 1 or DI ≠ 1) and versus histology (ND-OPMD or D-OPMDs/OSCCs) were evaluated by 2 by 2 contingency table analysis. By using this approach we found that 6 CNAs were associated with DNA aneuploidy and 2 CNAs were associated with a DNA diploid status in ND-OPMDs (Table 3), whereas in D-OPMDs/OSCCs, 21 CNAs were associated with DNA aneuploidy and none with DNA diploid status (Table 3). Concerning the relationship between CNAs and histology of the samples, 12 CNAs were associated with D-OPMDs/OSCCs and 1 with ND-OPMDs (Table 4).

**Table 3:** Association of CNAs with ploidy status in OPMDs/OSCCs. P-values and q-values of the associations are the indicated.

CAN	Diploid (DI = 1) OPMDs/OSCCs without CNAs	Diploid (DI = 1) OPMDs/OSCCs with CNAs	Aneuploid (DI ≠ 1) OPMDs/OSCCs without CNAs	Aneuploid (DI ≠ 1) OPMDs/OSCCs with CNAs	CNA-ploidy prevalence	p-value	q-value
			**ND-OPMDs**				
8p gain	35	0	7	4	DI ≠ 1	0.0020	0.0089
8p11.23 gain	35	0	7	4	DI ≠ 1	0.0020	0.0089
14q32.33 gain	10	25	9	2	DI = 1	0.0036	0.0198
8q gain	34	1	7	4	DI ≠ 1	0.0088	0.0268
19q loss	35	0	8	3	DI ≠ 1	0.0109	0.0268
19p loss	35	0	8	3	DI ≠ 1	0.0109	0.0268
11p15.5 gain	15	20	9	2	DI = 1	0.0376	0.0613
15q11.1 gain	32	3	7	4	DI ≠ 1	0.0458	0.0613
			**D-OPMDs/OSCCs**				
20q gain	26	0	13	9	DI ≠ 1	0.0003	0.0027
20p gain	25	1	12	10	DI ≠ 1	0.0011	0.0051
20q13.33 gain	17	9	4	18	DI ≠ 1	0.0014	0.0051
2q11.2 gain	24	2	12	10	DI ≠ 1	0.0059	0.0145
9p21.3 loss	24	2	12	10	DI ≠ 1	0.0059	0.0145
9p13.3 gain	25	1	14	8	DI ≠ 1	0.0071	0.0203
8q gain	23	3	12	10	DI ≠ 1	0.0111	0.0234
3p loss	26	0	17	5	DI ≠ 1	0.0154	0.0245
1p32.2 gain	26	0	17	5	DI ≠ 1	0.0154	0.0245
4q35.1 loss	25	1	15	7	DI ≠ 1	0.0167	0.0245
1q44 gain	25	1	15	7	DI ≠ 1	0.0167	0.0245
13q loss	25	1	15	7	DI ≠ 1	0.0167	0.0245
13q32.1 loss	25	1	15	7	DI ≠ 1	0.0167	0.0245
1q42.13 gain	24	2	14	8	DI ≠ 1	0.0293	0.0369
2q22.1 loss	26	0	18	4	DI ≠ 1	0.0376	0.0387
2q gain	26	0	18	4	DI ≠ 1	0.0376	0.0387
2p gain	25	1	16	6	DI ≠ 1	0.0376	0.0387
5p gain	25	1	16	6	DI ≠ 1	0.0376	0.0387
16q24.3 gain	19	7	9	13	DI ≠ 1	0.0395	0.0438
16p13.3 gain	15	11	6	16	DI ≠ 1	0.0447	0.0438

**Table 4:** Association of CNAs with sample histology. P-values and q-values of the associations are the indicated.

CAN	ND-OPMDs without CNAs	ND-OPMDs with CNAs	D-OPMDs/OSCCs without CNAs	D-OPMD/OSCCs with CNAs	CNA-histology prevalence	p-value	q-value
4q35.1_loss	46	0	40	8	D-OPMD/OSCC	0.0057	0.0665
13q_loss	46	0	40	8	D-OPMD/OSCC	0.0057	0.0665
5p_gain	46	0	41	7	D-OPMD/OSCC	0.0124	0.0665
20p_gain	44	2	37	11	D-OPMD/OSCC	0.0145	0.0665
7q_gain	45	1	39	9	D-OPMD/OSCC	0.0156	0.0665
9p13.3_gain	45	1	39	9	D-OPMD/OSCC	0.0156	0.0665
9p21.3_loss	43	3	36	12	D-OPMD/OSCC	0.0224	0.0747
9p_loss	46	0	42	6	D-OPMD/OSCC	0.0266	0.0747
4q_loss	46	0	42	6	D-OPMD/OSCC	0.0266	0.0747
14q11.2_gain	39	7	47	1	ND-OPMD	0.0288	0.0747
13q32.1_loss	45	1	40	8	D-OPMD/OSCC	0.0307	0.0747
1q44_gain	45	1	40	8	D-OPMD/OSCC	0.0307	0.0747
3q27.1_gain	43	3	37	11	D-OPMD/OSCC	0.0404	0.0762

### Relationship between CNAs and anatomical subsite: BM or TNG OPMDs and OSCCs

To verify whether some CNAs were differentially found between high- and low risk oral mucosa subsites in patients affected by OPMDs/OSCCs [42], we analyzed the distribution of the 80 CNAs identified by GISTIC2.0 between TNG and BM. In fact, these are sites at high and low risk of cancer development, respectively [42].

The results showed that 3 CNAs, namely the 8q, 8q24.3 and 20q13.33 gains were associated with TNG OPMDs and OSCCs (Table 5). Stratified analysis for the two histological groups (ND-OPMDs and D-OPMDs/OSCCs) performed for each of these three CNAs did not show any significant differences in odds-ratios (BD tests not significant). Furthermore, the 8q, 8q24.3 and 20q13.33 gains appeared to be associated with DNA aneuploidy (P-values: 4.6E-03, 9.0E-03, 3.6E-03, respectively), while only the 8q gain was associated with DNA aneuploidy in ND-OPMDs (P-value = 2.1E-02). These associations were all significant for multiple test correction at q-value < 0.1. It is noteworthy that the 8q24.3 and the 20q13.33 gains frequently co-occurred in the same samples and they showed a very high correlation (Cohen’s Kappa = 0.886, Pearson’s Chi-Square P = 1.1E-13).

**Table 5:** CNAs associated with TNG or BM OPMDs/OSCCs. P-values and q-values of the associations are the indicated.

CAN	BM OPMDs/OSCCs without CNAs	BM OPMDs/OSCCs with CNAs	TNG OPMDs/OSCCs without CNAs	TNG OPMDs/OSCCs with CNAs	CNA-site prevalence	p-value	q-value
20q13.33_gain	20	7	15	28	TNG	0.0029	0.0303
8q24.3_gain	19	8	14	29	TNG	0.0030	0.0377
8q_gain	26	1	30	13	TNG	0.0064	0.0508

Additionally, when we focused our analysis on ND-OPMDs we found that the 14q32.33 gain was associated with TNG ND-OPMDs (P-value = 2.9E-03, q-value = 1.6E-02) and with DNA diploid rather than DNA aneuploid ND-OPMDs (P = 9.7E-03). In this case, stratified analysis showed a statistically significant difference between the two histological groups (ND-OPMDs and D-OPMDs/OSCCs) (BD test P = 6.3E-3).

### Relationship between total focal and broad CNAs and histology in OPMDs and OSCCs

**Fig 3:**
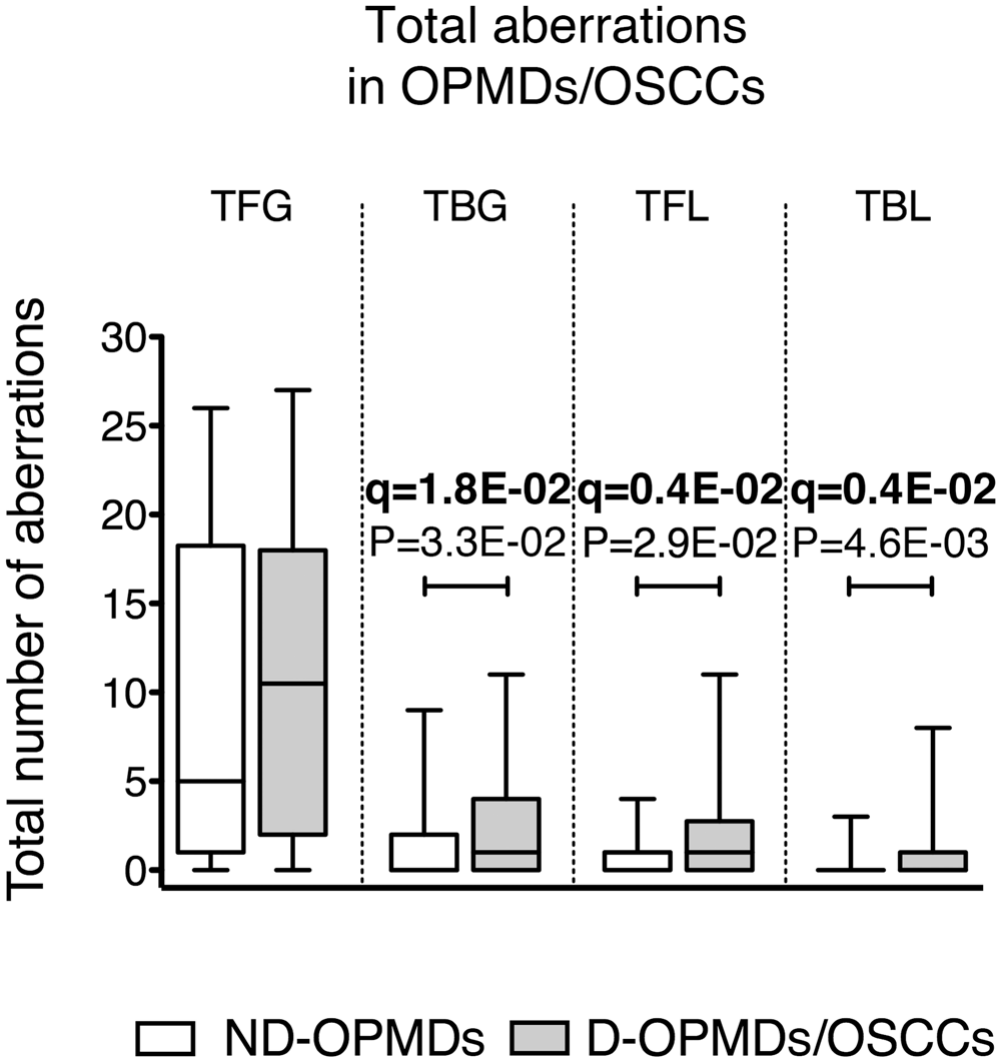
Relationship between total CNAs and histological diagnosis in OPMDs/OSCCs. The bottom and the top of each box show the first and third quartile, respectively, while the line inside the box represents the median (second quartile). Please notice that when the median is not shown, its value = 0. The tips of the whiskers represent the minimum and the maximum data value. Oral potentially malignant disorders (OPMDs); oral squamous cell carcinomas (OSCCs); non-dysplastic oral potentially malignant disorders (ND-OPMDs); dysplastic oral potentially malignant disorders (D-OPMDs). CNAs are referred to as: total focal gains, TFG; total broad gains, TBG; total focal losses, TFL; total broad losses, TBL. Broad gains and broad losses correspond to gains or losses of more than half a chromosome arm, respectively. The boxes corresponding to the number of CNAs detected in ND-OPMD sites are shown in white; the boxes corresponding to the number of CNAs detected in mucosa sites affected by D-OPMDs and OSCCs are shown in gray. Significant MW P-values (P < 0.05) and their corresponding q-values are shown. The FDR q-value method was applied for multiple testing (n = 4) correction; q-values < 0.1 are indicated in bold. N = 94 OPMDs/OSCCs; N = 46 ND-OPMDs; N = 48 D-OPMDs/OSCCs.

To determine whether the total DNA damage in terms of CNAs was differently distributed between the two histology groups (ND-OPMD versus D-OPMD/OSCC), the CNA data identified by the GISTIC analysis were processed as described in the Materials and Methods section, and the TFG, TBG, TFL and TBL were calculated for each histology group (Fig 3). Statistical analysis performed with the non-parametric MW two-tailed test showed that TBG, TFL and TBL were significantly higher in the D-OPMDs/OSCCs (P = 3.3E-02, P = 2.9E-02 and P = 4.6E-03, respectively; 95% CI) (Fig 3).

### Relationship between total focal and broad CNAs and DNA ploidy in OPMDs and OSCCs

**Fig 4:**
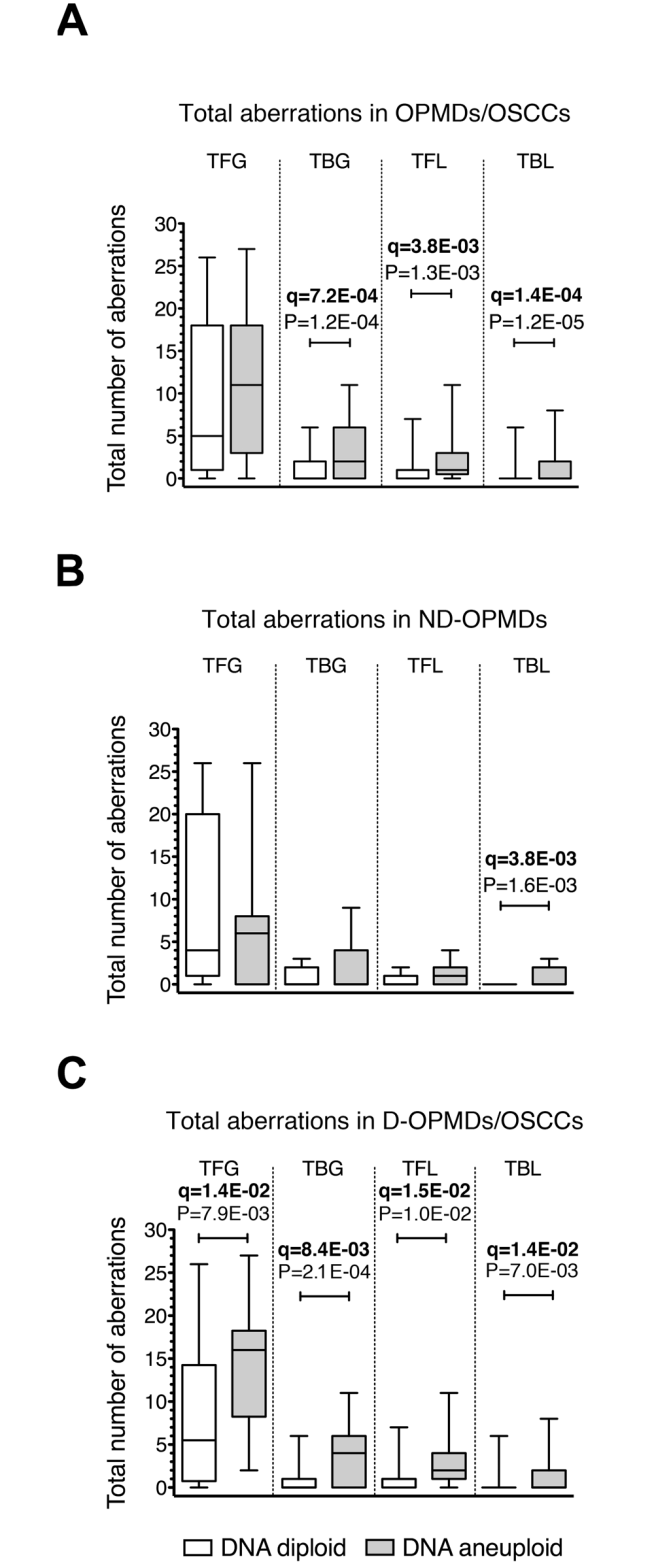
Relationship between total CNAs and DNA ploidy in OPMDs/OSCCs. (A) shows the number of total CNAs detected in oral potentially malignant disorders (OPMDs) and oral squamous cell carcinomas (OSCCs); (B) shows the number of total CNAs detected in non-dysplastic OPMDs (ND-OPMDs); (C) shows the number of total CNAs detected in dysplastic OPMDs (D-OPMDs) and OSCCs. The bottom and the top of each box show the first and third quartile, respectively, while the line inside the box represents the median (second quartile). Please notice that when the median is not shown, its value = 0. The tips of the whiskers represent the minimum and the maximum data value. The boxes corresponding to the number of CNAs detected in DNA diploid sites of oral mucosa are shown in white; the boxes corresponding to the number of CNAs detected in DNA aneuploid sites of oral mucosa are shown in gray. CNAs are referred to as: total focal gains, TFG; total broad gains, TBG; total focal losses, TFL; total broad losses, TBL. Broad gains and broad losses correspond to gains or losses of more than half a chromosome arm, respectively. Significant MW P-values (P < 0.05) and their corresponding q-values are shown. The FDR q-value method was applied for multiple testing (n = 4) correction; q-values < 0.1 are indicated in bold. (A) N = 94 OPMDs/OSCCs; N = 58 DNA diploid; N = 36 DNA aneuploid. (B) N = 46 ND-OPMDs; 35 DNA diploid; 11 DNA aneuploid. (C) N = 48 D-OPMDs/OSCCs; 24 DNA diploid; 24 DNA aneuploid.

To assess the distribution of chromosomal aberrations in OPMDs and OSCCs between the two DNA ploidy categories, total DNA damage scores were again explored using the non-parametric Mann-Whitney statistic. TBG and both TFL and TBL were significantly associated with aneuploid OPMDs/OSCCs (MW test P = 1.2E-04, P = 1.3E-03 and P = 1.2E-05, respectively) (Fig 4A). However, when the ND-OPMDs were analyzed separately, we observed that only the TBL was significantly associated with the DNA aneuploid ND-OPMDs (MW test P = 1.6E-03) (Fig 4B). On the other hand, each of the four total CNAs types we considered was significantly associated with DNA aneuploid D-OPMDs and OSCCs (MW test P = 7.9E-03, TFG; P = 2.0E-04, TBG; P = 1.0E-02, TFL; P = 7.0E-03, TBL) (Fig 4C).

### Relationship between total focal and broad CNAs and OPMD/OSCC mucosa subsites

**Fig 5:**
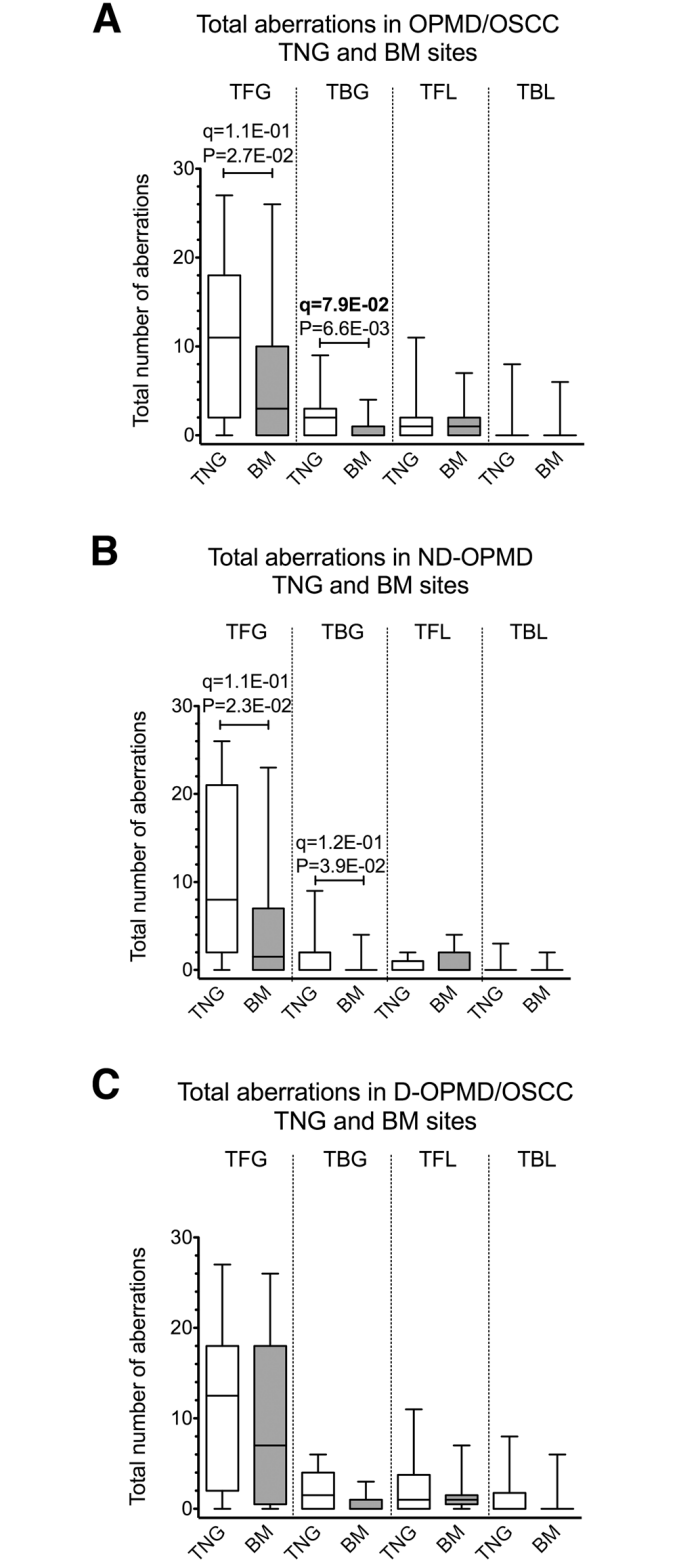
Relationship between total CNAs and histological diagnosis in TNG and BM OPMDs/OSCCs. (A) shows the number of total CNAs detected in oral potentially malignant disorder (OPMD) and oral squamous cell carcinoma (OSCC) in tongue (TNG) and buccal mucosa (BM) sites; (B) shows the number of total CNAs detected in non-dysplastic OPMDs (ND-OPMDs) in TNG and BM sites. (C) shows the number of total CNAs detected in dysplastic OPMDs (D-OPMDs) and OSCC TNG and BM sites. The bottom and the top of each box show the first and third quartile while the line inside the box represents the median (second quartile). Please notice that when the median is not shown, its value = 0. The tips of the whiskers represent the minimum and the maximum data value. CNAs are referred to as: total focal gains, TFG; total broad gains, TBG; total focal losses, TFL; total broad losses, TBL. Broad gains and broad losses correspond to gains or losses of more than half a chromosome arm, respectively. The boxes corresponding to the number of CNAs detected in TNG OPMDs/OSCCs are shown in white bars, whereas those of CNAs detected in BM OPMDs/OSCCs are shown in gray bars.

To verify whether the overall genomic damage was differently distributed in the oral mucosa subsites that were taken into consideration, we measured the total number scores for focal and broad CNA gains and losses separately (see MM for details) for both TNG and BM subsites (the most frequent ones in our CGH dataset, Table 1). The analysis showed that the total number of both focal and broad CNA gains was higher in TNG compared to BM OPMDs/OSCCs (Fig 5A). A similar result was obtained when only ND-OPMDs were analyzed (Fig 5B). However, it must be pointed out that after correcting for multiple testing, only the association between TBG and TNG in OPMDs/OSCCs reached our fixed threshold (q-value = 0.079) (Fig 5A). Lastly, no differences were observed in the total CNA distribution between TNG and BM D-OPMDs/OSCCs (Fig 5C).

Significant MW P-values (P < 0.05) and their corresponding q-values are shown. The FDR q-value method was applied for multiple testing (n = 4) correction; q-values < 0.1 are indicated in bold. A) N = 70 OPMDs/OSCCs; N = 43 TNG; N = 27 BM OPMDs/OSCCs. B) N = 37 ND-OPMDs; N = 19 TNG; N = 18 BM. C) N = 33 D-OPMDs/OSCCs; N = 24 TNG; N = 9 BM.

## Discussion

We investigated the relationship between genomic DNA aberrations in terms of CNAs as detected by aCGH, DNA aneuploidy or DI ≠ 1 as obtained by hr DNA-FCM, the histology of the OPMD/OSCC and the oral subsite where the OPMD/OSCC was located. We found that CNAs and DNA aneuploidy represent early events during the transition from ND-OPMDs to D-OPMDs and OSCCs.

In particular, we observed an increasing proportion of DNA aneuploidy along the sequence from ND-OPMD to D-OPMD to OSCC, thus confirming previous results [13, 14, 18].

Our present data refine and extend our previous observation of a strong correlation of DNA aneuploidy with OPMDs arising on TNG mucosa [43]. Here, we show that both ND-OPMDs and D-OPMDs/OSCCs limited to TNG mucosa were more frequently associated with DNA aneuploidy than those limited to BM. The rate of DNA aneuploidy was similar between TNG and BM in patients affected by OPMDs/OSCCs at multiple oral subsites. This finding suggests that ND-OPMDs limited to TNG may represent a clinical condition at higher risk of cancer development compared to ND-OPMDs in patients with multiple oral mucosa subsite involvement that includes TNG.

Our study also showed that the occurrence of specific CNA gains and losses that were absent or detected at a low frequency in ND-OPMDs increased in D-OPMDs/OSCCs. We suggest that the CNAs in this group may play a role in the transition from ND-OPMD to D-OPMD/OSCC. In particular, we hypothesize that the 1q44, 9p13.3, and 20p gains and the 9p21.3 and 13q32.1 losses which were present at a low frequency in ND-OPMD and at a higher frequency in D-OPMDs/OSCCs and that were also associated with DNA aneuploidy may help to identify ND-OPMDs at higher risk of progression. It should be highlighted that the 9p13.3 and 20p gains were frequently detected in low-grade OPMDs that subsequently progressed to invasive OSCC [44], and that they were reported at a high frequency in head and neck OSCCs [45], respectively. It is noteworthy that the LOH at the 9p21.3 region was reportedly linked with alterations in the INK4a/ARF locus which frequently precede the onset of oral cancer [46]. Interestingly, we observed that the 9p21.3 deletion was the most frequent focal loss in ND-OPMD, although its absolute proportion was lower in this disorder (about 6%) compared to the higher frequency (25%) in D-OPMD/OSCC, as previously reported [47]. Lastly, we found a 1q44 gain and a 13q32.1 loss which have never previously been reported in squamous cell carcinoma.

We hypothesize that the 5p gain and the 4q35.1 and 13q losses, which were present at low frequency in D-OPMDs/OSCCs but were never detected in the present study in ND-OPMDs, and that were also associated with DNA aneuploidy could represent markers of high risk of oral epithelial transformation for the D-OPMDs. Interestingly, deletion of the tumor suppressor gene *inhibitor of growth family*, *member 2*, located at the 4q35.1 region was associated with advanced tumor stage in head and neck SCCs [48].

One possible explanation for our finding of the 14q11.2 gain associated with ND-OPMDs is that this CNA might be unfavorable to the transition to the dysplastic-transformed state.

Our aCGH analysis also showed the preferential association of the 8q, 8q24.3, and 20q13.33 gains with TNG OPMDs/OSCCs and DNA aneuploidy. Furthermore, this analysis suggests that the 8q, 8q24.3, and 20q13.33 gains could play a specific role in TNG cancer onset and progression. The meaning of the high frequency of co-occurrence of the 8q24.3 and 20q13.33 CNA gains in OPMDs/OSCCs remains to be established. It should be pointed out that previous reports showed that chromosome 8 CNAs are common alterations in oral cancers [49] and that *MYC* amplification, whose locus is contained in the 8q24.3 region, plays an oncogenic role [50]. Interestingly, the 8q24.3 gain was previously associated with lymph node extra-capsular spread, development of second primary malignancies and poor survival in OSCC [51]. In addition, the 20q13.33 gain was identified in head and neck cancers [52] as well as in OPMDs and in normal looking mucosa fields distal to OPMDs [14]. At the moment it is not clear whether the 8q, 8q24.3, and 20q13.33 gains may help in the early identification of high risk ND-OPMDs. On the other hand, the association of the 14q32.33 gain with TNG ND-OPMDs and DNA diploid status suggests that this CNA may identify TNG lesions at low risk of epithelial transformation. To the best of our knowledge, the 14q32.33 gain was not commonly detected in cancer and it likely represents a copy number variation (CNV) that segregates in prostate cancer patients in high-risk African families [53]. Further studies are required to establish whether the 14q32.33 gain represents a germline CNV or a CNA associated with a different subgroup of OPMDs/OSCCs from those carrying other CNA gains.

An analysis of total CNAs in OPMDs/OSCCs clearly showed that TBG, TFL and TBL were higher in D-OPMDs/OSCCs compared with ND-OPMDs, whereas no significant differences in the extent of TFG were found between the two histological groups. These data might suggest that TBG, TFL and TBL are more closely related to the development of CIN and to the transition from non-dysplastic to dysplastic disorders and cancer. An analysis of the relationship between total CNAs and DNA aneuploidy showed a significant correlation between all the types of CNAs we considered and DNA aneuploidy in D-OPMDs and OSCCs, whereas a significant correlation was only found for the TBL in ND-OPMDs. These results are in agreement with a suggested role for broad CNA losses in promoting cancer development [54] and the existence of a link between CIN, loss of heterozygosity and tumorigenesis [55, 56]. However, we believe that a much larger number of samples needs to be examined before a definitive conclusion can be drawn about the difference in TBL between DNA diploid and DNA aneuploid ND-OPMDs.

The aCGH analysis performed in this study demonstrated a statistical significance for the higher burden of TBG that we observed in TNG OPMDs/OSCCs compared to those originating from the BM, which is the most frequent OPMD subsite. These data and the preferential association of the 8q, 8q24.3, and 20q13.33 gains and of DNA aneuploidy with TNG compared to BM OPMDs/OSCCs reported herein strongly suggest that these two oral mucosa subsites follow different pathways of CIN, e.g. high rate CIN vs. low rate CIN [57], respectively, during cancer development.

## Conclusions

Our study shows that: i) DNA aneuploidy, genomic damage (measured as total number of CNAs) and specific focal CNAs occur early during the development of oral cancer and become more frequently found at later stages; ii) OPMDs limited to TNG mucosa display a higher frequency of DNA aneuploidy compared to OPMDs limited to BM mucosa; iii) TNG OPMDs/OSCCs display peculiar features of genomic instability compared to BM OPMDs/OSCCs, given the preferential association with total broad and specific focal CNA gains.

A large follow-up study is clearly needed to determine whether specific CNAs and DNA aneuploidy may help to predict OSCC development in patients with ND-OPMDs.

## Supporting Information

S1 Table Focal CNAs identified in our sample set by GISTIC2.0. (XLSX)

S2 Table Broad CNAs identified in our sample set by GISTIC2.0 analysis. (XLSX)
